# Differential Left Hippocampal Activation during Retrieval with Different Types of Reminders: An fMRI Study of the Reconsolidation Process

**DOI:** 10.1371/journal.pone.0151381

**Published:** 2016-03-18

**Authors:** Cecilia Forcato, Luz Bavassi, Gabriela De Pino, Rodrigo Sebastián Fernández, Mirta Fabiana Villarreal, María Eugenia Pedreira

**Affiliations:** 1 Laboratorio de Neurobiología de la Memoria, Departamento de Fisiología, Biología Molecular y Celular, Instituto de Fisiología, Biología Molecular y Neurociencias-CONICET, Facultad de Ciencias Exactas y Naturales, Universidad de Buenos Aires, Buenos Aires, Argentina; 2 Fundación para la Lucha contra las Enfermedades Neurológicas de la Infancia, CONICET, Buenos Aires, Argentina; 3 Centro Universitario de Imágenes Médicas, Escuela de Ciencia y Tecnología, Universidad Nacional de San Martín, Buenos Aires, Argentina; 4 Departamento de Física, Facultad de Ciencias Exactas y Naturales, Universidad de Buenos Aires, Argentina; Hangzhou Normal University, CHINA

## Abstract

Consolidated memories return to a labile state after the presentation of cues (reminders) associated with acquisition, followed by a period of stabilization (reconsolidation). However not all cues are equally effective in initiating the process, unpredictable cues triggered it, predictable cues do not. We hypothesize that the different effects observed by the different reminder types on memory labilization-reconsolidation depend on a differential neural involvement during reminder presentation. To test it, we developed a declarative task and compared the efficacy of three reminder types in triggering the process in humans (Experiment 1). Finally, we compared the brain activation patterns between the different conditions using functional magnetic resonance imaging (fMRI) (Experiment 2). We confirmed that the unpredictable reminder is the most effective in initiating the labilization-reconsolidation process. Furthermore, only under this condition there was differential left hippocampal activation during its presentation. We suggest that the left hippocampus is detecting the incongruence between actual and past events and allows the memory to be updated.

## Introduction

Memories are dynamic rather than static. After being stored, they can be modified through further experience. Thus, inactive consolidated memories can be reactivated (labilized) through the presentation of cues (reminders) that were presented during acquisition. This presentation results in memory labilization, followed by a process of re-stabilization known as reconsolidation [1,2]. An operative definition of memory reconsolidation generally includes the assertion that the reactivated memory can be disrupted by an interfering agent such as protein synthesis inhibitors, β-blockers, or also a new learning process [3]. Furthermore, not all cues are equally effective in making the memories labile again [4–6]. Unpredictable cues are effective in triggering the labilization-reconsolidation process; however, predictable cues are not [7–9]. Thus, the discrepancy (mismatch) between what it is predicted according to previous stimuli contingency and what actually happens during reminder presentation determines if a memory trace will become labile or not [7–9].

In a previous study we analyzed the reminder predictability [3], participants learned a list of syllable pairs (cue-syllable associated to a response-syllable) linked to a specific context (color light, image and music) on day 1. On day 2, they received different types of reminders followed by a second learning task to interfere the re-stabilization of the labilized memory. Finally, subjects were tested on day 3. We found that the reconsolidation process was only triggered after a specific reminder structure formed by the context plus one cue-syllable. On the contrary, if only the context or one entire syllable pair was presented as reminder we did not observed labilization of the memory.

The reconsolidation process has been described in humans from a behavioral and pharmacological point of view. However, only few studies investigated the neural correlates for the reconsolidation process using functional magnetic resonance imaging (fMRI). Schwabe et al (2012) administered the β-adrenergic receptor antagonist propranolol to healthy participants before they reactivated previously learned neutral and emotional material. The results showed that propranolol during reactivation, specifically reduced the subsequent memory for emotional pictures, and diminished the activation of the amygdala [10]. Using a Pavlovian conditioning it has been demonstrated that an extinction training during reconsolidation prevents the return of fear and inactivates a memory trace in the basolateral amygdala. Furthermore, this treatment modifies the circuitry which underlies this process diminishing prefrontal cortex involvement [11,12]. Schacter et al (2013), studying neutral memories generated during a museum tour, revealed that the recognition of tour's events were better when the memories were highly reactivated than when they were reactivated at a lower level, although this high-reactivation also increases memories' distortions (false recognition). The fMRI results also revealed that the quality of reactivation modulated subsequent true and false memories via the recruitment of left parahippocampal bilateral retrosplenial, and bilateral posterior inferior parietal cortices [13].

Based on our previous results, we hypothesize that the beginning of the labilization-reconsolidation process depends on specific features of the retrieval, therefore different neural correlates will be associated with different types of reminders. To test this hypothesis, we developed a learning task (picture-word associations) that shares similarities with the syllable pair protocol but with the possibility of analyzing the reactivation period inside an fMRI scanner. The task involves different types of reminders: context reminder, which includes only the image; the syllable reminder which includes the image plus the cue (first syllable of the word); and the word reminder which is formed by the image and the complete word. The different reminder-structures are similar to that used in our previous paradigm [3], allowing us to compare between retrieval and retrieval plus reactivation. As in the other learning task, we expect that the unpredictability emerges when the subjects cannot perform the expected action, learned it during training (mismatch, [7]). During the training session participants have to complete the associated word only after the appearance of the image plus the first syllable. Therefore, we predict that the syllable reminder generates a mismatch (a prediction error) triggering the labilization process as the reminder is interrupted before volunteers can answer. In this report, we performed two experiments. In the first experiment (Experiment 1) we compared the efficacy of three different reminders in triggering the labilization-reconsolidation process. In Experiment 2, using fMRI, we analyzed brain activation patterns during the reminders presentation. Furthermore, we expect that processing a declarative memory might evoke activity in the hippocampus [14]. Particularly, the left hippocampus is activated during the retrieval of the incomplete item [14–16]. In this experimental design this situation occurred during the syllable reminder presentation. Thereby, we analyzed the left hippocampal activity between reminders using region of interest analysis. As we predicted, only the reminder that included the mismatch (the unpredictable event) induced the labilization-reconsolidation process and only in this condition there was a differential left hippocampal activation pattern during the presentation of the reminder.

## Materials and Methods

### Subjects

96 healthy undergraduate and graduate students from Buenos Aires University volunteered for the study (56 women, 40 men). Their ages ranged from 18 to 35 years, with a mean of 25±1. 50 subjects participated in the behavioral experiments (Experiment 1), and 46 subjects participated in the second experiment that included fMRI (Experiment 2). The data from 11 subjects were excluded from the behavioral analysis because they did not reach more than fifty percent of correct responses at training. The data from 7 subjects were excluded from the fMRI analysis: 1 subject fell asleep, another repeatedly closed his eyes during the fMRI acquisition, and 5 subjects moved during acquisition. The criteria used for movement’s tolerance was up to 2 mm, which corresponds to the size of a voxel and a half, of the original acquisition dimension.

Before their participation in the experiments, the participants signed a written informed consent form. Both, the protocol and the consent were previously approved by the Ethics Committee of the Fundación para la Lucha contra las Enfermedades Neurológicas de la Infancia (FLENI), in accordance with the declaration of Helsinki.

### Experimental Procedure

As it was mentioned in the Introduction, we performed two experiments. In Experiment 1 we tested the efficacy of our paradigm in triggering the labilization-reconsolidation process. In Experiment 2 we analyzed brain activation patterns during this paradigm using fMRI.

Basically, the experiments were performed in three days, each separated by 48 hours. On day 1 subjects learned a list of pictures-words associations (Training). On day 2 the list were divided in three conditions depending on the reminders type (Reactivation). After this, a subgroup of subjects was randomly selected to learn a second list of pictures-words associations (Interference task). On day 3, all subjects were tested (Testing). The responses were quantified according the type of reminders.

#### Training

The learning task consisted of associating 36 pictures with 36 Spanish words (i.e. picture of sky associated to the word “PALOMA”, pigeon). The words were nouns, and each noun had three syllables and six letters. Each word started with a different syllable (S1 Fig). The pictures-words (items) were not directly related in a semantic dimension, they did not share the same category. This part of the experiment was performed on a computer as it is described below in the Experimental Setup.

Each picture was presented for 3 seconds followed by the designated word overlaid for 1 second, and then a black screen was shown for 4 seconds. This sequence was repeated until all the 36 items were presented.

**Fig 1:**
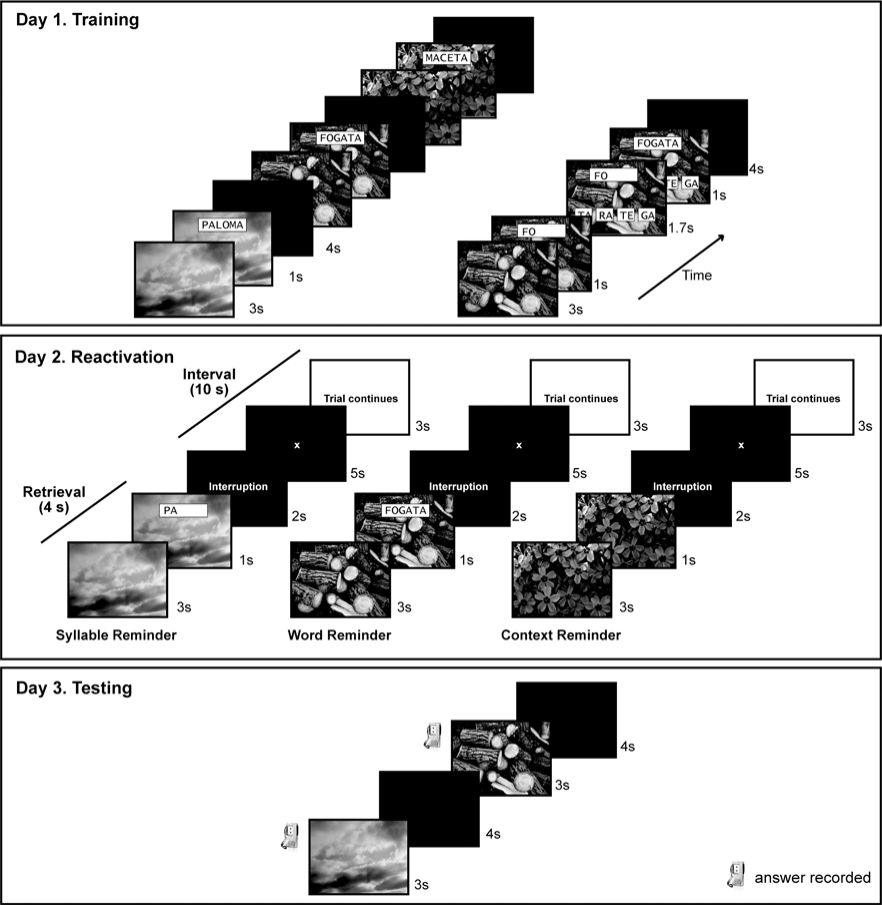
Experimental Design.

Next the items were evaluated The picture was shown for 3 seconds but then only the first syllable of the associated word was overlaid for 1 second (for example, a picture of the sky and the first syllable of the word “PALOMA” = PA). At this moment, four possible options of the two correct syllables that complete the entire word appeared at the bottom of the screen. The subjects had 1.7 seconds to press the two keys that would complete the word. The keyboard had four keys available to answer with the right hand. The first key represented the first option on the monitor’s screen, the second key represented the second option, and so on. If the subjects answered correctly, the word stayed in black for 1 second; if the subjects answered incorrectly, or did not respond on time, the correct answer appeared in red for 1 second (Fig 1). The training lasted 15 minutes. All the answers were recorded (See Fig 1 Day 1 Training).

#### Reactivation

All the 36 items were divided in three conditions or reminders (context, syllable and word), pseudorandomly assigned for each participant. The reminders consisted on the presentation of the following sequence: 1) a picture during 3 seconds, 2) a cue for one second, 3) a message of interruption for 2 seconds, 4) a red cross for fixation for 5 seconds, 5) a message indicating that the trial will continue for 3 seconds. The difference between the three reminders was set during the cue step. In the syllable reminder, the cue was the first syllable of the word displayed superimposed with the picture. In the word reminder the cue was the entire word over the picture; and in the context reminder the cue was the picture itself (Fig 1 Day 2 Reactivation). This sequence was repeated until all the 36 items were shown, and immediately after that, the sequence was repeated one more time until all the 36 items were presented again, but this second time in a different order with respect to the first time. It is important to emphasize that the options for completing the word never appeared during the reactivation task. The reactivation lasted 17 minutes. Before the beginning of the reactivation all the subjects received the same instruction.

**The Instruction:** Now, all the picture-word associations will be presented. The pictures will appear followed by the first syllable of the associated word and the four options to answer. Every time you see the first syllable of the word you have to complete the word according to the options given on the screen.

#### Interfering task

The interfering task involved learning a second list of picture-words (with different pictures and words) using the same protocol as the training. The words had the same first syllable than the words in training (S1 Fig).

#### Testing

During testing, each picture was presented for 3 seconds, and the subjects had to say the associated word aloud. The inter-trial interval was 4 seconds (Fig 1 Day 3 Testing). All 36 items were tested and it lasted 7.2 minutes. The answers of the participants were recorded.

### Type of experiments and experimental groups

Two separate experiments were performed.

#### Experiment 1 (n = 39)

It was conducted in an experimental room at the University of Buenos Aires and included three groups.

**Reactivation group (n = 13):** subjects were trained (day 1), they received the reactivation (day 2) and were tested (day 3).

**Reactivation/interference group (n = 13):** subjects were trained (day 1), they received the reactivation followed by the interference task (day 2) and were tested (day 3).

**No-reactivation group (n = 13):** subjects were trained (day 1) and tested (day 3). In this group the results of the testing were divided randomly in three categories emulating the 3 types of reminders.

Subjects were assigned to one of the three groups in accordance with their performance at training, in order to maintain an homogeneous distribution between groups.

#### Experiment 2 (n = 39)

Subjects were trained and tested in the experimental room used for Experiment 1, but the reactivation was performed during fMRI acquisition in the scanner at the FLENI Institute. The interference task was performed in another experimental room at the FLENI Institute.

**Reactivation group (n = 14):** as in Experiment 1.

**Reactivation/interference group (n = 10):** as in Experiment 1.

To study the brain activity associated with the reminder presentation the fMRI data of the reactivation and reactivation/interference group were analyzed together (Trained group, n = 24).

**Untrained group (n = 15):** subjects received only the reactivation, without being trained or tested to see the effect of stimulus-related processing of the reactivation without memory intervention.

All participants received the same instruction in the reactivation session. Groups were created applying the same criteria that in Experiment 1.

Table 1 summarizes the groups of each Experiment.

**Table 1:** Experimental groups.

Experiment 1	n	Experiment 2	n
*Reactivation group*	13	*Reactivation group*	14
*Reactivation/interference group*	13	*Reactivation/interference group*	10
*No-reactivation group*	13	*Untrained group*	15

### Experimental setup

The paradigm was designed and presented using homemade software in Matlab 7.5 (Mathworks Inc., Sherborn, MA, USA) with the Psychtoolbox toolkit [17–19]. During the reactivation in the scanner, the subjects laid comfortably supine inside the bore of the magnet, with their heads fixed to minimize movement. The paradigm was projected on a screen from a computer outside the scanner room, which the subjects could see through a mirror mounted on the head coil. They were provided with a keyboard with 4 buttons and were instructed to press the keys to complete the words based on the options displayed.

### fMRI data and image processing

#### Image acquisition

A 3-Tesla General Electric Signa HDxt (GE Medical Systems, Milwaukee, Wisconsin, USA) scanner was used to acquire all the images. An 8-channel head coil was used for reception of the signal intensity. A three-plane localizer image was initially obtained to facilitate the positioning of the transverse sections parallel to the anterior-posterior commissure line. For fMRI, an interleaved T2*-weighted gradient echo EPI sequence was used to cover the whole brain (TR/TE = 2500/30 ms; acquisition matrix size = 64x64; FOV = 24 cm; slice thickness = 4 mm, with zero spacing between images; an in-plane resolution of 3.75x3.75 mm^2^; and 30 contiguous sections). The total acquisition time was 17 minutes, including 5 dummy scans to allow for T1 saturation effects that were discarded from the analysis. A total of 408 volumes were acquired. For anatomic reference, a high resolution T1-weighted 3D fast SPGR-IR was used (TR = 6.604 ms, /TE = 2.796 ms, /TI = 450); parallel imaging (ASSET) acceleration factor = 2; acquisition matrix size = 256x256; FOV = 24 cm; slice thickness = 1.2 mm; 120 contiguous sections).

#### Image Processing

Data were analyzed using Statistical Parametric Mapping (SPM5, Wellcome Department of Cognitive Neurology, University College, London, UK) implemented in MATLAB (Mathworks Inc., Sherborn, MA, USA). A slice-timing correction was applied to each volume. The imaging time series was realigned to the first image to correct for the subject´s motion during acquisition and spatially normalized using Montreal Neurological Institute reference brain [20]. The spatially normalized volumes consist of 2 mm^3^ voxels. These data were subsequently smoothed with an isotropic Gaussian kernel of 8 mm at full width half-maximum [21].

Our main interest was to analyze the response during the three types of reminders, Thus we modeled each one (composed of the picture plus the cue, 4 secs) with the canonical hemodynamic response function, leaving as regressors of no interest the interval between reminders (interruption plus fixation cross plus message of trial continues: 10 secs) grouped together, and the 6 parameters for the head movement corrections. This analysis was performed individually over the data of the 24 participants (Trained group: Reactivation and Reactivation/interference groups, see Table 1).

For this whole brain fMRI statistical analysis we performed individual contrasts between the different reminder types (Syllable > Word; Syllable > Context and Word > Context) for each subject. Then, those contrasts were taken into a second level analysis (one sample t-test) in order to find the group response. Results are shown at a threshold of p<0.05 FWE (family wise error, corrected for multiple comparisons). The same analysis was performed for the untrained group (see Table 1).

**ROIs Analysis:** Besides the individual whole brain analysis we quantified the left hippocampal activity using a mask of this region obtained from the AAL Atlas [22]. We calculated the percentage of signal change (β-values) for all the subjects of Experiment 2 (see Table 1). To perform the analysis we used MarsBaR (MARSeille Boîte À Région d’Intérêt) [23].

We performed a repeated measures 2 x 3 ANOVA, with group as the between-subjects factor (with two levels: trained and untrained groups) and reminder type as the within-subjects factor (with three levels: context, syllable and word reminders), using a threshold of α = 0.05 [24]. In the cases we obtained a significant group per condition interaction, simple effects analysis was performed followed by pairwise comparisons corrected by Bonferroni’s adjustment for multiple comparisons (α = 0.05).

### Statistical Analysis for the Behavioral Data

#### Experiment 1

The percentage of correct responses at training and testing was analyzed by repeated measures ANOVA, with group as the between-subjects factor (with three levels: reactivation, reactivation/interference and no-reactivation groups) and reminder type as the within-subjects factor (with three levels: context, syllable and word reminders), using a threshold of α = 0.05 [24]. When a significant group per condition interaction was detected, we performed simple effects analysis followed by pairwise comparisons corrected by Bonferroni’s adjustment for multiple comparisons (α = 0.05).

#### Experiment 2

The percentage of correct responses at training and at testing was analyzed by repeated measures 2 x 3 ANOVA, with the group as the between-subjects factor (with two levels: reactivation and reactivation/interference) and reminder type as the within-subjects factor (with three levels: context, syllable and word reminders), using a threshold of α = 0.05 [24]. When a significant group per condition interaction was detected, we performed simple effects analysis followed by pairwise comparisons corrected by Bonferroni’s adjustment for multiple comparisons (α = 0.05).

## Results

### Experiment 1

**Fig 2:**
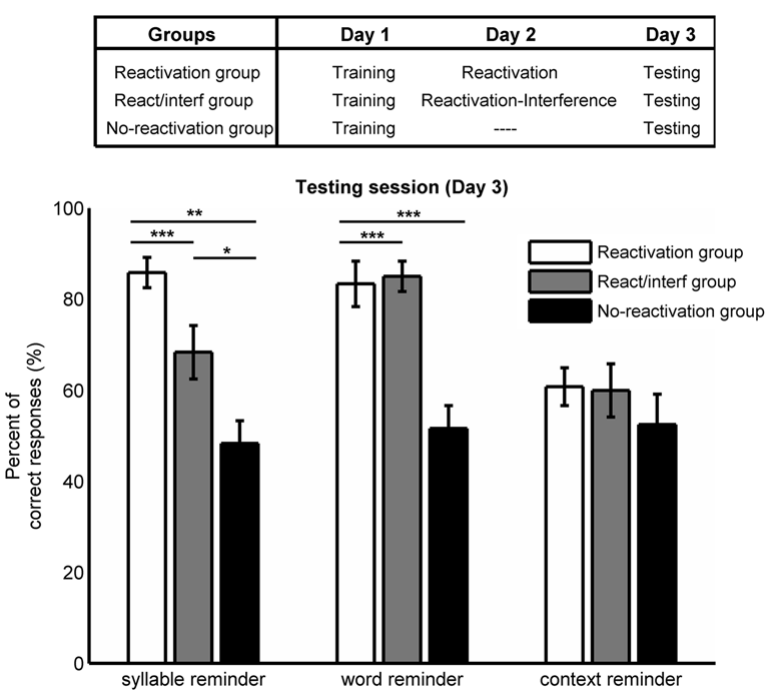
Experiment 1. Mean percentage of correct responses at testing session ± SEM, for the different groups and types of reminders. *, p<0.05; **, p<0.01 and ***, p<0.001.

The goal of this experiment was to evaluate the efficacy of three different types of reminders on triggering memory labilization-reconsolidation (Fig 2). A repeated measures ANOVA revealed a significant group per reminder type interaction (F(4,72) = 6.989, p<0.001). Thus, we performed simple effects analyses of group within each reminder type followed by pairwise comparisons corrected by Bonferroni’s adjustment for multiple comparisons. There was a significant simple effect of group within syllable reminder (F(2,36) = 17.331, p<0.001). On one hand, for the syllable reminder (picture plus first syllable of the associated word), the reactivation group showed significantly higher percentage of correct responses than the reactivation/interference group at testing (p = 0.032), while the no-reactivation group had a significantly smaller percentage of correct responses than the other groups (reactivation vs. no-reactivation p<0.001; reactivation/interference vs. no-reactivation p = 0.009. Reactivation group: 86±3; reactivation/interference group: 68±6; no-reactivation group: 48±5). For the word reminder (picture plus designated word), there was a significant simple effect of group within word reminder type (F(2,36) = 18.035, p<0.001). There was no significant difference between the reactivation and reactivation/interference group at testing (p = 1.000). However, the no-reactivation group showed a significantly lower percentage of correct responses compared with the other groups (both p<0.001; reactivation group: 83±5; reactivation/interference group: 85±3; no-reactivation group: 52±5). For the context reminder (picture alone), we found no significant difference between groups (simple effects of group within context reminder F(2,36) = 0.924, p = 0.400; reactivation group: 61±4; reactivation/interference group: 60±6; no-reactivation group: 53±7. 06).

On the other hand, we observed that in the reactivation group the syllable and the word reminders did not differ in the percentage of correct responses at testing whereas the context reminder produced significantly fewer percentage of correct responses than the syllable and word reminders (simple effects of reminder type within reactivation group, F(2,35) = 14.553, p<0.001; Bonferroni’s adjustment for multiple comparisons for syllable vs. word reminder p = 1.000; for syllable reminder vs. context reminder p<0.001; and for word vs. context reminder p = 0.002). Furthermore, the no-reactivation group showed no significant differences in the percentage of correct responses between reminders suggesting that different combinations of picture-noun associations were equally remembered at testing (simple effects of reminder type within no-reactivation group, F(2,35) = 0.154, p = 0.858).

In addition, there were no significant differences in the percentage of correct responses between groups at training neither for reminder type (Repeated measures ANOVA, groups F(2,36) = 0.289, p = 0.751; reminder types F(2,72) = 2.768, p = 0.070; interaction F(4,72) = 0.810, p = 0.523, left panel S2 Fig).

These results show that the syllable reminder was effective in triggering memory labilization-reconsolidation. This process was revealed by a second learning task, which interfered the re-stabilization of the memory trace.

### Experiment 2

To explore the brain activity associated with the reminder presentation we performed a similar behavioral experiment but the reactivation was conducted in an fMRI scanner.

The behavioral results at testing showed the same profile as in Experiment 1 (S3 Fig). Thus, performing the reactivation in different contexts (Experiment 1 in the experimental room; and Experiment 2 in the fMRI scanner) did not modify the efficacy of the reminders in triggering labilization-reconsolidation.

**Fig 3:**
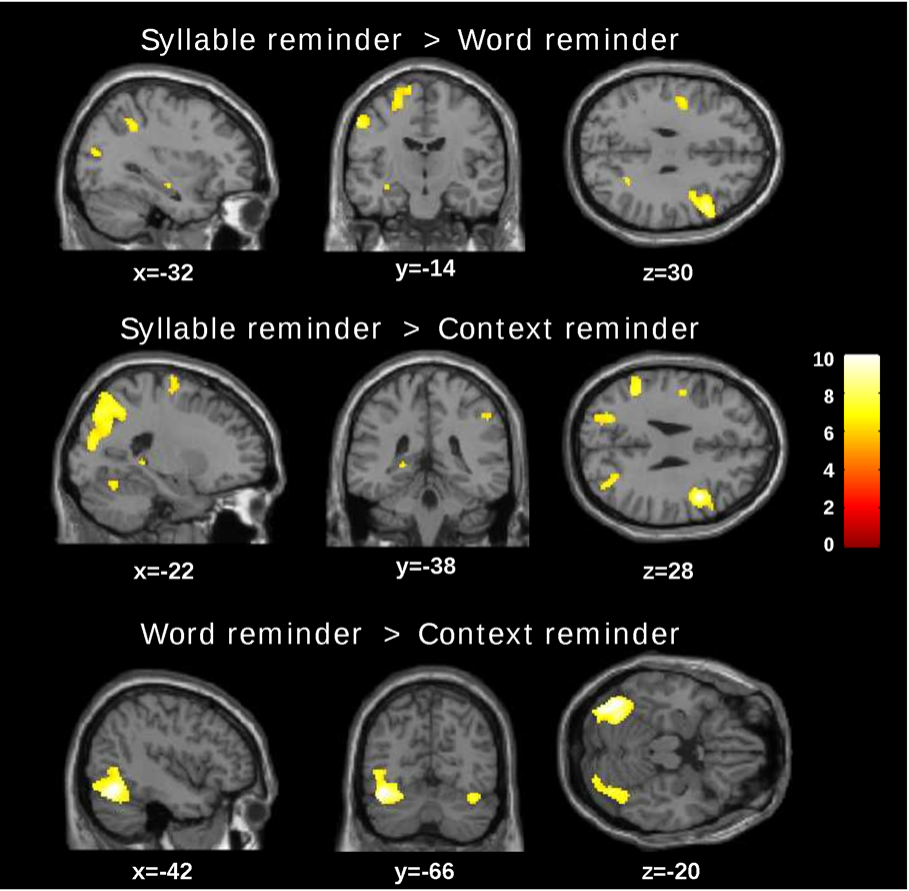
t-maps. Comparison between reminder conditions for the trained group (p<0.05 FWE corrected p-value, cluster size).

Firstly, we compared the brain activity during the reminder presentation between the three reminder conditions. Because this presentation occurred before the interference task we decided to group the fMRI data of the reactivation and reactivation/interference group together (trained group). Fig 3 shows the t-maps of the different comparisons while S1 Table summarizes the significant differences. Specifically, the syllable reminder showed a higher left hippocampal activity in both comparisons: syllable reminder > word reminder: (Voxel FWE corrected p = 0.041 T- = 6.27); syllable reminder > context reminder (Voxel FWE corrected p = 0.018, T- = 6.65). Additionally, we found significant activation of areas involved in different sensory processing, executive control and working memory tasks (S1 Table) [25–28]. A similar analysis comparing the word and context reminder showed higher activation in the left fusiform, the right fusiform and the right rolandic operculum areas for the word reminder (word reminder>context reminder). We found no significant differences in any brain region in the opposite comparisons (word reminder > syllable reminder, context reminder > syllable reminder and context reminder > word reminder.

To corroborate that the differences in the brain activity between reminders were due to mnemonic processes and not to stimulus-related processing without memory intervention, we run a control group (untrained group) in which subjects received only the reactivation without the training. Applying the same whole brain analysis there were no significant differences in hippocampal activity in any of the comparisons(T<7.58, p(FWE corrected)>0.05, K = 0). However, we found significant differences in areas related to language and motor processing (S2 Table).

**Fig 4:**
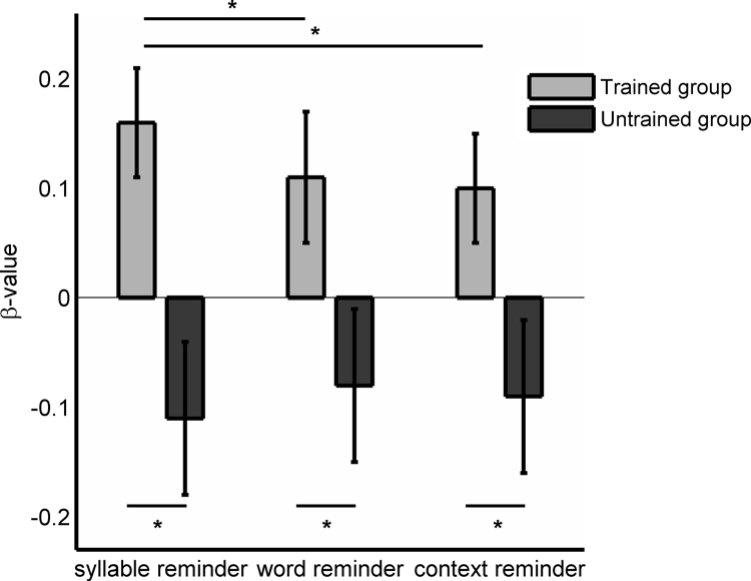
ROI analysis. β-values for the left hippocampal for the trained and untrained group.

For a deeper comparison between the trained and untrained groups, we quantified the left hippocampal activity based on regions of interest (MarsBars, see Materials and Methods). For the trained group we found significantly higher β-values for the syllable reminder than for the other reminder conditions, while the untrained group showed no significant differences between reminder types (Fig 4, Repeated measures ANOVA interaction: F(2,74) = 6.907 p = 0.002; simple effects of reminder type within trained group p = 0.002, Bonferroni’s adjustment for multiple comparisons: syllable vs. word reminder p = 0.002, syllable vs. context reminder p = 0.002 and word vs. context reminder p = 0.459. Simple effects of reminder type within untrained group p = 0.144). Furthermore, the three types of reminders showed significantly higher β-values for the trained compared to the untrained group (simple effect of group within syllable reminder p = 0.003; within word reminder p = 0.036 and within context reminder p = 0.038). Thus, the syllable reminder, which showed to be effective in triggering the labilization-reconsolidation process, showed higher left hippocampal activation during reactivation.

## Discussion

The reconsolidation process of a neutral declarative memory has been well described using behavioral and pharmacological approaches [3, 8, 29–31]. More recently, different reports investigated its neural correlates [10–12]. Here, we designed a task to study the retrieval during the reminder presentation associated with the labilization-reconsolidation process, using fMRI. We showed that only the reminder that included the unpredictable events (syllable reminder) triggered memory labilization-reconsolidation (Experiment 1) and only under this condition was asignificant enhanced of the activation of the left hippocampus. Specifically, this activation was a consequence of the mnemonic process and not a stimulus-related sensory processing (Experiment 2).

In this report, we demonstrated that only the syllable reminder labilized the picture-word associations leaving the memory susceptible to the interference task (Fig 2, syllable reminder for Reactivation group vs. Reactivation/ interference group). Interestingly, we found that the labilization-reconsolidation improved the memory 48 hours after reactivation (Fig 2, syllable reminder: reactivation vs. no reactivation groups), providing further evidence for one of the biological roles of memory reconsolidation: the memory strengthening [32, 33].

Moreover, we showed that neither the context reminder nor the word reminder had differences between the reactivation and reactivation/interference groups. One possible interpretation for these results is that the presentation of those reminders only retrieved the memory without initiating the labilization-reconsolidation process [3], leaving the memory intact and protected against interferences. The differential effects of the different reminder types on memory labilization-reconsolidation could be explained by a mismatch effect. That is to say, the discrepancy between what is predicted according to previous stimuli contingencies and actual facts could be driving the labilization-reconsolidation process [7,8]. On one hand, the word reminder consisted of the picture plus the entire associated word as in the training session. On the other hand, the syllable reminder consisted only of the picture plus the first syllable of the associated word and the trial was interrupted without any completeness of the word. The fact that the entire word was never presented in the syllable reminder, nor produced by the subject generated a mismatch that mediated memory labilization-reconsolidation. We have previously demonstrated in the syllable associate paradigm the importance of the reminder structure [3]. In that report, we showed that the mismatch was crucial for initiating the process and also that the omission of one component in the reminder structure–the cue syllable- could prevent memory labilization. Thus, we assume that the picture alone was not enough as cue to trigger memory labilization-reconsolidation.

Using fMRI, we determined the brain activity associated with the reminder presentation. The syllable reminder showed significantly higher left hippocampal activity than the other reminders (syllable reminder > word reminder; syllable reminder > context reminder, Fig 3 and S1 Table). This difference was related to the mnemonic process given the fact that there were no significant differences in the hippocampal activity in any comparisons of the control group. Finally, when we quantified the left hippocampal activity as region of interest we found higher beta activity for the syllable reminder and moreover, the untrained group showed similar β-values in the three conditions.

Declarative memory depends on the integrity of the medial temporal lobe [34] and the hippocampus is essential for declarative memory consolidation [14]. Subsequent studies have highlighted different roles for right and left hippocampus suggesting that the brain’s involvement in memory processes is lateralized. Looking for neural correlates, Kumaran and Maguire [15] used fMRI in a repetition paradigm in humans to evaluate whether the hippocampus provides neural representations of temporal order. The results revealed that the neural activity in the left hippocampus is related with the recognition of a partially rearranged object sequences from the exact one. In a further study, they observed similar results when the spatial location of the objects on the screen was partially changed [16].

In the present report, the increase in the left hippocampal activity for the syllable reminder could be related to the retrieval of the incomplete item [14], or as a consequence of the detection of the incongruence between the actual and learned events. We suggest that the mismatch is detected by the left hippocampus, which operates as a comparator opening a new time window that allows previous predictions to update when new information is detected.

## Supporting Information

S1 Fig Lists of pairs of associated picture-noun for the main task and for the Interference task. (TIF)

S2 Fig Training performance. Mean percentage of correct responses at training session ± SEM, for the different groups and reminder types.
(TIF)

S3 Fig Experiment 2. Mean percentage of correct responses at testing session ± SEM, for the different groups and reminder types.
(TIF)

S1 Table Comparison between reminders for the trained group. (DOCX)

S2 Table Comparison between reminders for the untrained group. (DOCX)
