# Supplementary figures and images for: Differential Left Hippocampal Activation during Retrieval with Different Types of Reminders: An fMRI Study of the Reconsolidation Process
Source: PLoS One. 2016 Mar 18;11(3):e0151381. doi: 10.1371/journal.pone.0151381 (PMC4798722; doi:10.1371/journal.pone.0151381)

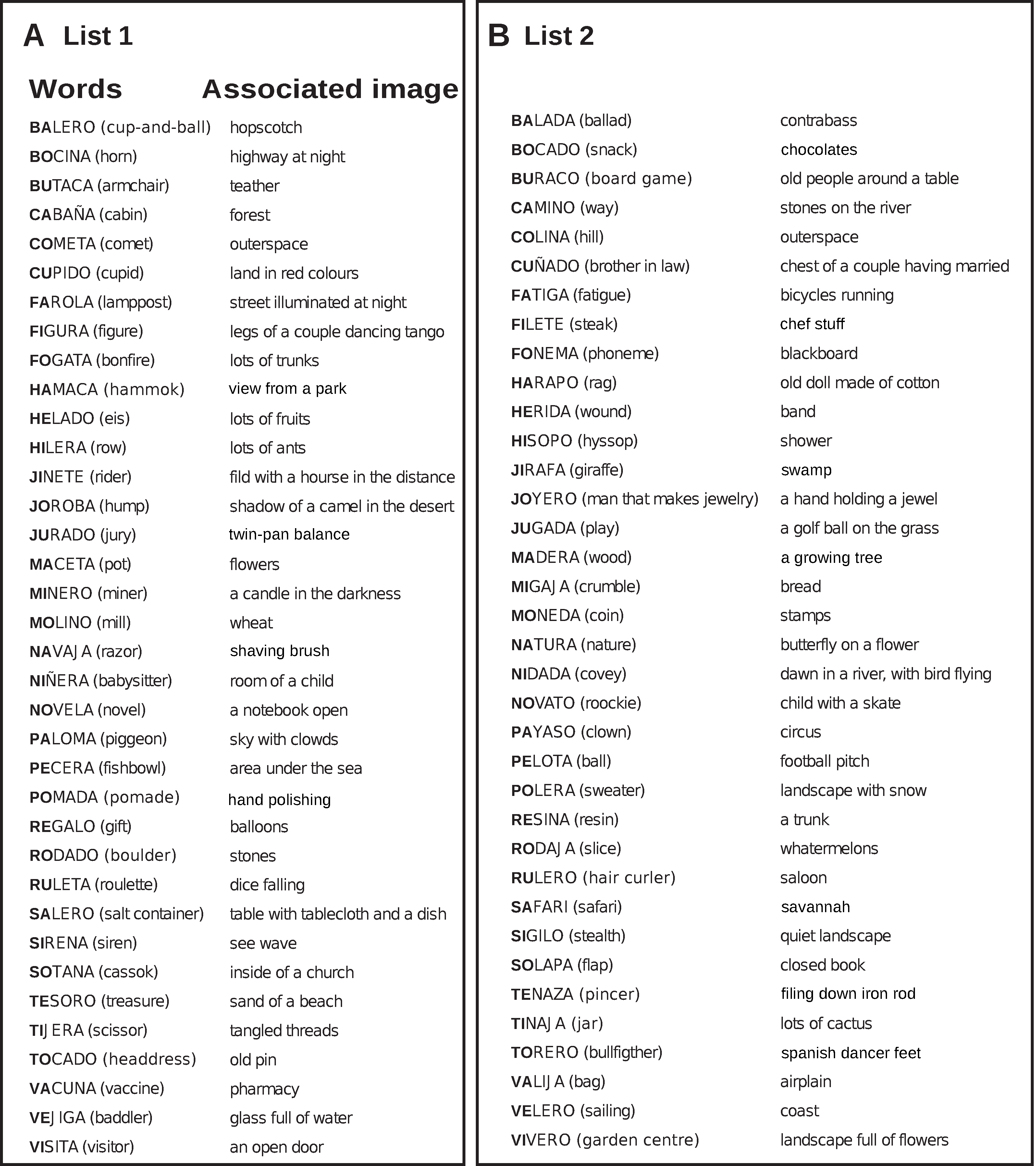

Supplement: S1 Fig — (TIF) [file pone.0151381.s001.tif]

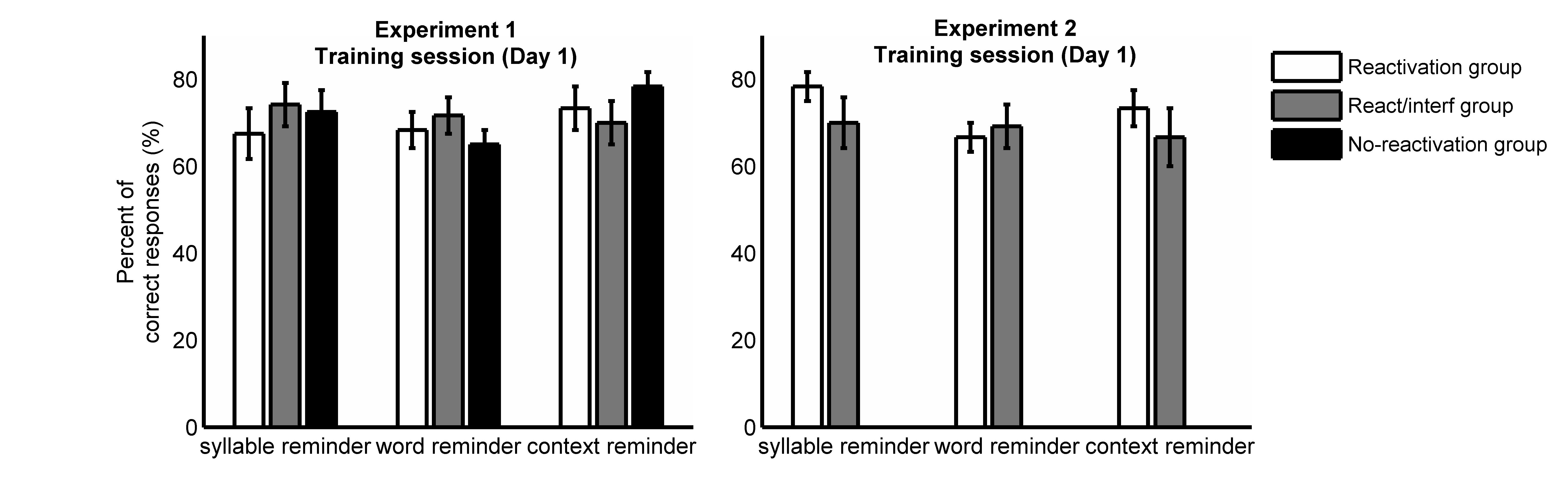

Supplement: S2 Fig — Mean percentage of correct responses at training session ± SEM, for the different groups and reminder types. (TIF) [file pone.0151381.s002.tif]

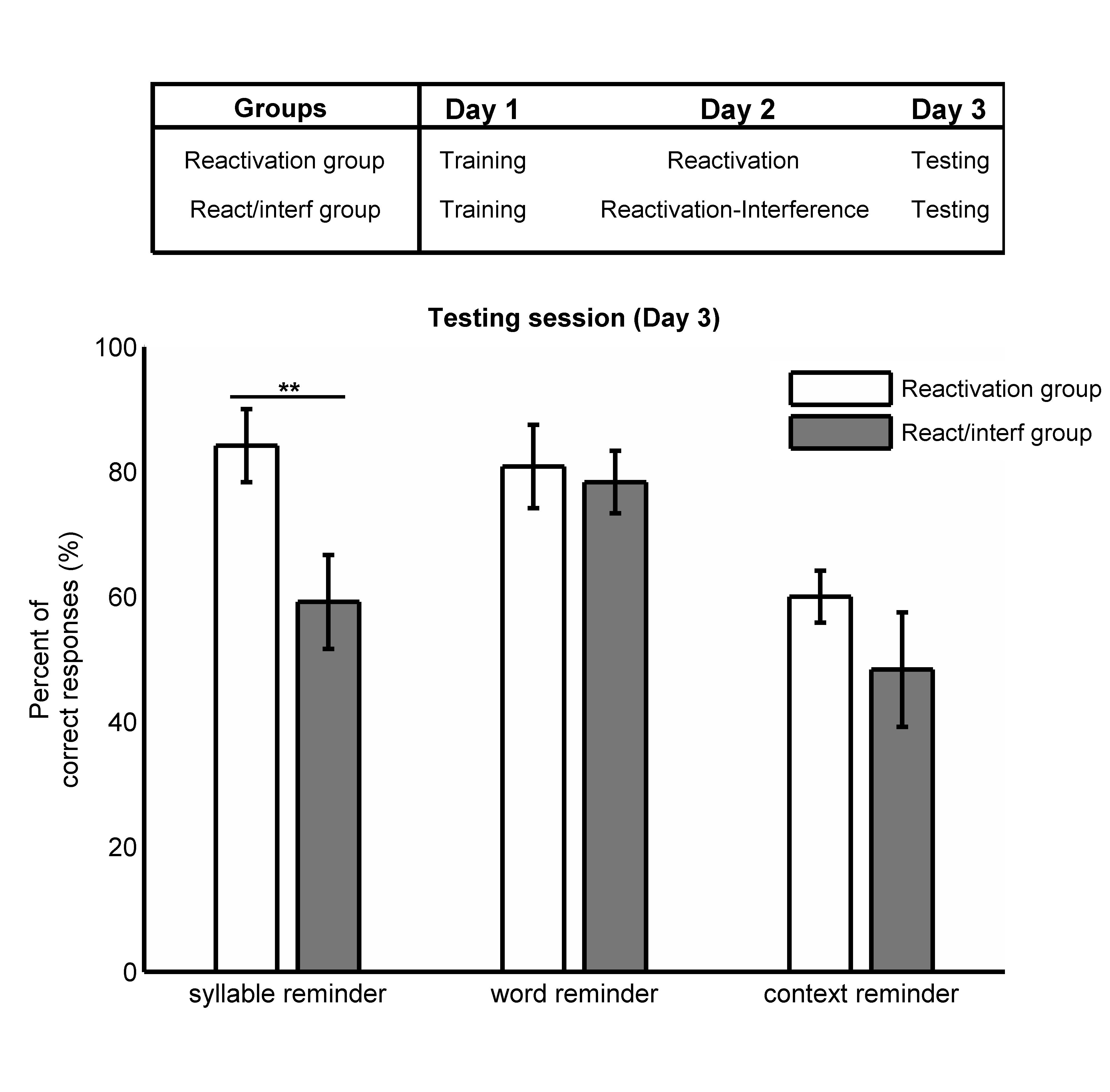

Supplement: S3 Fig — Mean percentage of correct responses at testing session ± SEM, for the different groups and reminder types. (TIF) [file pone.0151381.s003.tif]
