# Supplementary material for: Differential Left Hippocampal Activation during Retrieval with Different Types of Reminders: An fMRI Study of the Reconsolidation Process
Source: PLoS One. 2016 Mar 18;11(3):e0151381. doi: 10.1371/journal.pone.0151381 (PMC4798722; doi:10.1371/journal.pone.0151381)
Supplement: S1 Table — (DOCX) [file pone.0151381.s004.docx]

| **S1 TABLE.** | | | | | | | | | | | |
| --- | --- | --- | --- | --- | --- | --- | --- | --- | --- | --- | --- |
| **Brain Area** | **Cluster size**  **(n° voxels)** | **Cluster p**  **(corrected)** | | **Voxel**  **T-value** | **Voxel p(FWE-corrected)** | **MNI Coordinates (x,y,z)** | | | | | |
| **Syllable reminder > Word reminder** | | | | | | | | | | |  |
| **Left Supplementary Motor Area** | 562 | < 0.001 | | 8.87 | < 0.001 | | | -12 | -6 | 64 | |
| **Right Supplementary Motor Area** |  |  | | 7.97 | 0.002 | | | 2 | 2 | 62 | |
| **Left Superior Frontal** |  |  | | 7.50 | 0.004 | | | -24 | -8 | 68 | |
| **Right Inferior Frontal Operculum** | 281 | < 0.001 | | 8.56 | 0.001 | | | 50 | 16 | 30 | |
| **Right Precentral** |  |  | | 7.01 | 0.01 | | | 40 | 4 | 30 | |
| **Right Superior Parietal** | 363 | < 0.001 | | 8.43 | 0.001 | | | 26 | -70 | 48 | |
| **Left Postcentral** | 199 | < 0.001 | | 7.89 | 0.002 | | | -56 | -14 | 44 | |
| **Left Superior Parietal** | 502 | < 0.001 | | 7.81 | 0.002 | | | -14 | -70 | 58 | |
| **Left Precuneus** |  |  | | 7.70 | 0.003 | | | -12 | -70 | 50 | |
| **Left Inferior Parietal** |  |  | | 7.40 | 0.005 | | | -30 | -48 | 44 | |
| **Left Putamen** | 38 | 0.001 | | 7.37 | 0.005 | | | -18 | 4 | -8 | |
| **Right Precuneus** | 214 | < 0.001 | | 7.12 | 0.008 | | | 26 | -58 | 26 | |
| **Right Lingual** |  |  | | 7.06 | 0.009 | | | 8 | -54 | 4 | |
| **Left Caudate** | 28 | 0.001 | | 7.09 | 0.009 | | | -8 | 6 | 8 | |
| **Left Calcarine** | 43 | < 0.001 | | 7.01 | 0.01 | | | -20 | -66 | 10 | |
| **Left Middle Occipital** | 27 | 0.001 | | 6.84 | 0.014 | | | -34 | -80 | 16 | |
| **Right Inferior Orbitofrontal** | 12 | 0.006 | | 6.56 | 0.024 | | | 30 | 24 | -6 | |
| **Left Insula** | 7 | 0.012 | | 6.49 | 0.027 | | | -26 | 20 | -4 | |
| **Left Middle Temporal** | 3 | 0.022 | | 6.47 | 0.028 | | | -38 | -52 | 16 | |
| **Left Precentral** | 3 | 0.022 | | 6.29 | 0.039 | | | -46 | 0 | 44 | |
| **Left Hippocampus** | 3 | 0.022 | | 6.27 | 0.041 | | | -32 | -14 | -12 | |
| **Syllable reminder > Context reminder** | | | | | | | | | | | |
| **Right Middle Occipital** | 1466 | < 0.001 | | 11.54 | < 0.001 | | | 40 | -78 | 14 | |
| **Right Superior Occipital** |  |  | | 8.44 | 0.001 | | | 26 | -68 | 42 | |
| **Right Inferior Parietal** |  |  | | 7.71 | 0.003 | | | 34 | -50 | 42 | |
| **Left Middle Occipital** | 2124 | < 0.001 | | 10.45 | < 0.001 | | | -36 | -86 | 16 | |
| **Left Supramarginal** |  |  | | 8.28 | 0.001 | | | -48 | -48 | 26 | |
| **Left Inferior Occipital** | 1166 | < 0.001 | | 10.22 | < 0.001 | | | -46 | -70 | -16 | |
| **Left Middle Temporal** |  |  | | 7.12 | 0.007 | | | -50 | -60 | 0 | |
| **Right Inferior Frontal Operculum** | 744 | < 0.001 | | 9.64 | < 0.001 | | | 46 | 8 | 28 | |
| **Right Precentral** |  |  | | 7.67 | 0.003 | | | 50 | 4 | 40 | |
| **Left Postcentral** | 327 | < 0.001 | | 7.20 | 0.006 | | | -50 | -6 | 36 | |
| **Left Precentral** | 123 | < 0.001 | | 6.96 | 0.01 | | | -26 | -12 | 76 | |
| **Right Inferior Temporal** | 100 | < 0.001 | | 6.67 | 0.017 | | | 50 | -56 | -14 | |
| **Right Middle Temporal** |  |  | | 6.29 | 0.035 | | | 58 | -52 | -4 | |
| **Left Hippocampus** | 10 | 0.009 | | 6.65 | 0.018 | | | -22 | -38 | 2 | |
| **Right Middle Cingulum** | 5 | 0.018 | | 6.29 | 0.035 | | | 10 | -14 | 32 | |
| **Left Supplementary Motor Area** | 17 | 0.005 | | 6.29 | 0.035 | | | -10 | -8 | -66 | |
| **Right Supramarginal** | 8 | 0.012 | | 6.25 | 0.038 | | | 52 | -38 | 44 | |
| **Right Supplementary Motor Area** | 6 | 0.015 | | 6.14 | 0.046 | | | 4 | 2 | 64 | |
| **Word reminder > Context reminder** | | | | | | | | | | | |
| **Left Fusiform** | 1485 | | < 0.001 | 10.78 | < 0.001 | | -42 | | -66 | -20 | |
| **Right Fusiform** | 324 | | < 0.001 | 8.29 | 0.001 | | 26 | | -84 | -16 | |
| **Right Rolandic Operculum** | 13 | | 0.008 | 6.42 | 0.026 | | 58 | | 6 | 16 | |
