# Supplementary material for: Differential Left Hippocampal Activation during Retrieval with Different Types of Reminders: An fMRI Study of the Reconsolidation Process
Source: PLoS One. 2016 Mar 18;11(3):e0151381. doi: 10.1371/journal.pone.0151381 (PMC4798722; doi:10.1371/journal.pone.0151381)
Supplement: S2 Table — (DOCX) [file pone.0151381.s005.docx]

| **S2 TABLE.** | | | | | | | | | |
| --- | --- | --- | --- | --- | --- | --- | --- | --- | --- |
| **Brain Area** | **Cluster size**  **(n° voxels)** | **Cluster p**  **(corrected)** | **Voxel**  **T-value** | **Voxel p(FWE-Corrected)** | **MNI Coordinates (x,y,z)** | | | | |
| **Word reminder > Context reminder** | | | | | | | | |  |
| **Right Pallidum** | 59 | < 0.001 | 11.77 | 0.002 | | 14 | -2 | 4 | |
| **Left Frontal Inferior Triangularis** | 361 | < 0.001 | 11.34 | 0.004 | | -54 | 24 | 2 | |
| **Left Frontal Inferior Operculum** |  |  | 10.78 | 0.007 | | -52 | 12 | 2 | |
| **Left Middle Temporal** | 40 | <0.001 | 10.86 | 0.006 | | -50 | -48 | 8 | |
| **Left Inferior Temporal** | 75 | < 0.001 | 10.35 | 0.01 | | -50 | -66 | -6 | |
| **Left Precentral** | 15 | < 0.001 | 8.17 | 0.07 | | -38 | 6 | 30 | |
| **Left Fusiform** | 34 | < 0.001 | 8.08 | 0.07 | | -42 | -58 | -16 | |
| **Syllable reminder > Context reminder** | | | | | | | | |  |
| **Left Superior Parietal** | 13 | < 0.001 | 8.43 | 0.05 | | -24 | -64 | 52 | |
| **Left Precentral** | 13 | < 0.001 | 8.41 | 0.05 | | -42 | -10 | 62 | |
